# Sperm Lipid Composition in Early Diverged Fish Species: Internal vs. External Mode of Fertilization

**DOI:** 10.3390/biom10020172

**Published:** 2020-01-22

**Authors:** Kathrin M. Engel, Viktoriya Dzyuba, Alexandre Ninhaus-Silveira, Rosicleire Veríssimo-Silveira, Dirk Dannenberger, Jürgen Schiller, Christoph Steinbach, Borys Dzyuba

**Affiliations:** 1 Institute for Medical Physics and Biophysics, Medical Faculty, University of Leipzig, Härtelstr. 16–18, 04107 Leipzig, Germany; juergen.schiller@medizin.uni-leipzig.de; 2 Faculty of Fisheries and Protection of Waters, University of South Bohemia in Ceske Budejovice, South Bohemian Research Centre for Aquaculture and Biodiversity of Hydrocenoses, Zátiší, 728/II, 38925 Vodňany, Czech Republic; vdzyuba@frov.jcu.cz (V.D.); steinbach@frov.jcu.cz (C.S.); bdzyuba@frov.jcu.cz (B.D.); 3 Department of Biology and Zootechny, Ilha Solteira, Faculty of Engineering, São Paulo State University, Neotropical Ichthyology Laboratory—LINEO, Monção Street, 226, 15385-000, Ilha Solteira, SP, Brazil; alexandre.ninhaus@unesp.br (A.N.-S.); rosicleire.verissimo@unesp.br (R.V.-S.); 4 Leibniz Institute for Farm Animal Biology, Institute of Muscle Biology and Growth, Lipid Metabolism and Muscular Adaptation Workgroup, Wilhelm-Stahl-Allee 2, 18196 Dummerstorf, Germany; dannenberger@fbn-dummerstorf.de

**Keywords:** lipidomics, sperm, freshwater fish, mass spectrometry, thin-layer chromatography, fertilization mode

## Abstract

The lipid composition of sperm membranes is crucial for fertilization and differs among species. As the evolution of internal fertilization modes in fishes is not understood, a comparative study of the sperm lipid composition in freshwater representatives of externally and internally fertilizing fishes is needed for a better understanding of taxa-specific relationships between the lipid composition of the sperm membrane and the sperm physiology. The lipidomes of spermatozoa from stingray, a representative of cartilaginous fishes possessing internal fertilization, and sterlet, a representative of chondrostean fishes with external fertilization, have been studied by means of nuclear magnetic resonance (NMR), matrix-assisted laser desorption/ionization time-of-flight mass spectrometry (MALDI-TOF MS), electrospray MS, gas chromatography-(GC) MS, and thin-layer chromatography (TLC). NMR experiments revealed higher cholesterol content and the presence of phosphatidylserine in stingray compared to sterlet sperm. Unknown MS signals could be assigned to different glycosphingolipids in sterlet (neutral glycosphingolipid Gal-Cer(d18:1/16:0)) and stingray (acidic glycosphingolipid sulpho-Gal-Cer(d18:1/16:0)). Free fatty acids in sterlet sperm indicate internal energy storage. GC-MS experiments indicated a significant amount of adrenic acid, but only a low amount of docosahexaenoic acid in stingray sperm. In a nutshell, this study provides novel data on sperm lipid composition for freshwater stingray and sterlet possessing different modes of fertilization.

## 1. Introduction

Fishes, representing a taxonomically not ranked group of animals, possess different modes of fertilization—internal and external. Internal fertilization in modern chondrichthyan fishes, which evolved from other groups of vertebrates [1] some 500 million years ago, was preserved from an ancient ancestor [2] of fishes, reptiles, birds, and mammals. Thus, internal fertilization in cartilaginous fishes has the same evolutionary origin as internal fertilization in reptiles, birds, and mammals. In modern actinopterygian fishes, which evolved from Osteichthyes [3] about 350 million years ago, the ancient internal mode of fertilization was substituted by an external one. Only the most ancient groups of Actinopteryhii (Cladistia and Chondrostei) have preserved some features of the evolutionary old internal mode of fertilization, e.g., the presence of the acrosome in spermatozoa. All these facts make fishes an attractive model for understanding the evolution of internal fertilization in vertebrates.

One of the essential aspects of the fertilization process is sperm motility activation, which may be significantly different in fishes with external and internal modes of fertilization. In externally fertilizing fishes, sperm motility activation is governed by specific factors including changes in environmental osmolality and ionic composition [4,5,6]. In internally fertilizing fishes, sperm transfer into the female reproductive tract occurs without noticeable changes in tonicity, but ionic factors seem to play a decisive role in sperm motility regulation [7]. Only first steps have been made so far to elucidate the mechanisms of sperm motility regulation in internally fertilizing fish species [8]. There could be two options for the state of spermatozoa inside the male reproductive tract: (I) spermatozoa are immotile, for example in some cases of teleostean fish species, such as the guppy [7] or the redtail splitfin [8], and (II) spermatozoa are motile, as it was recently shown for ocellate river stingray *Potamotrygon motoro* characterized by a reproductive system structure which resembles that of mammals rather than that of fishes [9].

Regardless of the fertilization mode, the mode of motility activation, the sensitivity of spermatozoa towards tonicity, and temperature changes, sperm motility is initiated in response to external signals acting at the level of the spermatozoon plasma membrane [6]. Nowadays, it can be stated that it is exactly the physicochemical state of the plasma membrane which determines reactions of fish spermatozoa to environmental changes occurring during fertilization. In carp, a representative of fish with an “osmotic” mode of sperm motility activation, sperm motility activation can be prevented by blocking mechano-sensitive channels whose activity is associated with the tension in the surrounding lipid bilayer [10]. In contrast, in trout and sterlet, spermatozoa do not significantly change their volume during motility acquisition [11]. These facts imply that the physiological reaction of spermatozoa to environmental changes is determined by a species-specific difference in the sperm plasma membrane lipid composition.

While the sperm lipid composition is known for many teleostean fish species with external fertilization, little is known about chondrostean fishes (external fertilization) and only very limited information is available for cartilaginous fishes (internal fertilization). To get a more detailed description of the cellular lipid composition, modern methods of lipidomics are required. Unfortunately, the sperm lipid composition of different teleostean and chondrichthyan fish species has so far only been studied using liquid chromatography (LC) and gas chromatography (GC) [12,13,14,15]. Analysis of the sperm membrane lipid composition using mass spectrometry (MS) and nuclear magnetic resonance (NMR) spectroscopy provides the most comprehensive information about differences in membrane structure and function between species. While NMR provides full (use of a standard is required) or relative quantitative information, information about the molecular (fatty acyl) compositions of all detected lipids can be obtained by MS.

For the present study, sperm of the externally fertilizing sterlet *Acipenser ruthenus* and the internally fertilizing ocellate river stingray *P. motoro* have been used as model objects representing chondrostean and cartilaginous fishes, respectively. The selection of these particular species was also inspired by the fact that both of them reproduce in fresh water. In this way, the potential effect of water salinity on sperm lipid composition was avoided. The sperm lipid compositions of sterlet and ocellate river stingray have been elucidated by means of MS, NMR, and high performance thin-layer chromatography (HPTLC) as shown for other externally fertilizing fish species only recently [16]. 

## 2. Materials and Methods 

### 2.1. Ethics

All manipulations of animals were performed in accordance with the authorization for the use of experimental animals (Reference number: 2293/2015-MZE-17214 (16OZ22302/2014-17214) valid from 22 January 2015 for five years) issued to the University of South Bohemia in Ceske Budejovice, Faculty of Fisheries and Protection of Waters (USB, FFPW) by the Ministry of Agriculture of the Czech Republic and approved by the local Ethics Commission on Animal Use (CEUA-FEIS/UNESP 14/2018). 

Fishing of *P. motoro* was done following the guidelines of a collecting permit issued by the Ministry of the Environment (MMA) of Brazil, Chico Mendes Institute for Biodiversity Conservation (ICMBio), Biodiversity Information and Authorization System (SISBIO) (no. 58102-1 issued on 31 March 2017).

### 2.2. Fish Rearing Conditions and Sperm Collection

Experiments were conducted using mature males of sterlet *A. ruthenus* (age: 4–6 years, weight: 1.2–1.9 kg, length: 49–61 cm) and ocellate river stingray *P. motoro* (age: unknown, weight: 1.0–1.9 kg, total length: 44–58 cm, disk width: 24–33 cm, disk length: 24–32 cm). Sterlet was reared at the hatchery of South Bohemian Research Center of Aquaculture and Biodiversity of Hydrocenoses, Vodnany, Czech Republic. According to common fisheries practice, spermiation in sterlet was stimulated by intramuscular injection of carp pituitary powder dissolved in aqueous 0.9% NaCl solution at 4 mg/kg body weight 24 h before sperm collection. Sperm was stripped by abdominal massage into dry collecting vials. 

Specimens of *P. motoro* were captured during natural spawning season (October–November) in the Paraná River with the assistance of local fishermen. The maintenance of the animals and collection of samples were carried out at the Laboratory of Neotropical Ichthyology (LINEO), Department of Biology and Zootechny, UNESP, Ilha Solteira, SP, Brazil. Following anesthesia with tricaine methane-sulfonate (MS 222), sperm samples were collected from cloaca following application of gentle pressure on the distal reproductive tract proximal to the cloaca and stored on ice before experimentation. 

For each fish species, sperm samples from five males were collected. Each stripped sperm sample was concentrated by centrifugation at 5000× *g* for 15 min at 4 °C. The obtained cell pellet was frozen at −80 °C until the time of lipid extraction [17].

### 2.3. Chemicals

All used solvents were obtained in the highest commercially available purity grade from Sigma-Aldrich (Taufkirchen, Germany) with the exception of deuterated solvents, which were purchased from Armar Chemicals (Döttingen, Switzerland). All chemicals were used as supplied.

### 2.4. Lipid Extraction

Sperm lipids were extracted according to the procedure by Bligh and Dyer [18] with slight modifications. Briefly, fish sperm were mixed with 4 mL methanol/chloroform (1:1, *v*/*v*) in glass vials, 2 mL aqua dest. were added for phase separation and batches were mixed vigorously. Samples were centrifuged for 10 min at 2500 rpm to achieve the separation into aqueous, protein-containing, and organic layers. The lower lipid-containing layer was withdrawn by a glass syringe and lipid extraction was repeated once more with an additional volume of 2 mL chloroform. Organic phases were combined. Aliquots of the organic phases were evaporated to dryness. 

### 2.5. Nuclear Magnetic Resonance

All NMR measurements were performed on a Bruker DRX 700 spectrometer (Bruker BioSpin GmbH, Rheinstetten, Germany) using a 5 mm TXI probe. For ^31^P NMR of phospholipids (PLs) the “mixed micelle” approach was used [19]. Dried lipid extracts (see Section 2.4) were dissolved in sodium cholate buffer (50 mM Tris, 200 mM sodium cholate, 5 mM EDTA, pH 7.65). Spectra were recorded in 5 mm NMR tubes at 283.3 MHz at 310 K. ^31^P measurements were performed with composite pulse decoupling (Waltz-16) to eliminate ^31^P-^1^H coupling. Pulse intervals of the order of T_1_ were used to allow relative quantitative analysis of PL integral intensities. Relative compositional data were considered to be sufficient because the extraction yields are significantly affected by the extraction method. Other parameters were acquisition time: 2 s; data size: 16 k; 60° pulse; pulse delay 3 s; and a line-broadening of 1 Hz.

For ^1^H NMR dried lipid extracts were dissolved in 0.5 mL CDCl_3_. Spectra were recorded at 300 K due to the reduced boiling point of chloroform compared to water. Spectra (16 k data points) were processed without any window functions, i.e., without Gauss or line broadening factor. Spectra were calibrated by setting the resonance of non-deuterated, residual CHCl_3_ to 7.24 ppm.

### 2.6. Matrix-Assisted Laser Desorption/Ionization Time-of-Flight Mass Spectrometry

Dried lipid extracts of fish sperm (see Section 2.4) were mixed 1:1 (*v*/*v*) with 0.5 M 2,5-dihydroxybenzoic acid (DHB) in methanol as matrix [20] for positive polarity and 10 mg/mL 9-aminoacridine (9-AA) in isopropanol/acetonitrile (60/40, *v*/*v*) for negative polarity [21] and vortexed for good homogeneity. A sample of 0.75 µL was transferred onto an aluminum-coated matrix-assisted laser desorption/ionization (MALDI) target (Bruker Daltonics GmbH, Bremen, Germany). MALDI-TOF spectra were recorded on a Bruker Autoflex “Speed” mass spectrometer which utilizes a 2 kHz solid-state laser emitting at 355 nm. For enhanced resolution and to average “cold” and “hot” spots spectra were recorded in the reflector mode by using the predefined laser firing algorithm “random walk”. The extraction voltage was 20 kV. Gated matrix suppression was applied to prevent saturation of the detector by matrix ions. For each mass spectrum 500 single laser shots were averaged. Laser-induced sample alterations were kept to a minimum by setting the laser energy only slightly above the threshold level. 

### 2.7. High Performance Thin-Layer Chromatography and Electrospray Ionization Mass Spectrometry

Dried lipid extracts were redissolved in chloroform and spotted onto a normal phase HPTLC glass plate (Merck KGaA, Darmstadt, Germany) with the help of a Linomat device (CAMAG, Muttenz, Switzerland). Plates were developed with chloroform/ethanol/water/triethylamine (30:35:7:35, by vol.) as the mobile phase [22] and lipids were visualized by dipping the entire plate in primuline (Direct Yellow 59, Sigma-Aldrich, Taufkirchen, Germany) (50 mg/L in acetone/water (80:20, *v*/*v*)). The lipids in each spot were automatically eluted by a Plate Express™ TLC plate reader (Advion, Ithaca, NY, USA) with methanol as solvent and directly transferred into the electrospray ionization ion trap ESI-IT mass spectrometer. 

ESI-IT MS was performed on an Amazon SL mass spectrometer (Bruker Daltonics GmbH) by direct infusion. The following conditions were used: Spray voltage 4.5 kV, end plate offset 500 V, nebulizer gas 7 psi, drying gas (N_2_) 3 L/min, capillary temperature 180 °C, flow rate 3 µL/min, sheath gas (He) flow rate 25 a.U. Spectra were recorded in the enhanced resolution mode by positive or negative ionization with a maximum ionization time of 50 ms. 

For the elucidation of the hexose moiety in selected sterlet sperm lipids the relevant spot was scratched off from the TLC plate and extracted with 2 mL methanol/chloroform (1:1, *v*/*v*) as described in Section 2.4. The dried extract was dissolved in methanol/chloroform (1:1, *v*/*v*) and automatically spotted onto a new HPTLC plate. C16 galactosyl(α) ceramide (d18:1/16:0) (Avanti Polar Lipids, Inc., Alabaster, AL, USA) was used as reference. The plate was developed with isopropanol/15 M NH_4_OH/aqua dest. (75:5:25, by vol.) as described in [23] and stained with primuline. 

### 2.8. Fatty Acid Analysis by Gas Chromatography

Dried lipid extracts were redissolved in chloroform/methanol (2:1, *v*/*v*) and a solution containing nonadecanoic acid (19:0) as the internal standard was added. A detailed sample preparation protocol for fatty acid (FA) derivatization was previously published [24]. FA analysis was performed using capillary GC with a CP-Sil 88 for FAME column (100 m × 0.25 mm, Agilent, Santa Clara, CA, USA) and a PerkinElmer gas chromatograph CLARUS 680 with a flame ionization detector and split injection (PerkinElmer Instruments, Waltham, MA, USA). The detailed GC conditions were described only recently [25]. The calibration was performed with the help of the reference standard mixture ‘Sigma FAME’ (Sigma-Aldrich, Deisenhofen, Germany) and the methyl esters of 18:1cis-11, 22:5n-3 and 18:2cis-9, trans-11 (Matreya LLC, State College, PA, USA), 20:3n-9, 22:3n-3 and 22:4n-6 (Sigma-Aldrich), and 18:4n-3 (Larodan, Limhamn, Sweden). The five-point calibration of single FAs ranged between 16 and 415 µg/mL and was checked after GC analysis of five samples.

### 2.9. Software

NMR spectra were processed using 1D WINNMR, version 6.2 (Bruker Analytische Messtechnik GmbH, Rheinstetten, Germany). MALDI-TOF MS data were acquired and processed by “Flex Control” and “Flex Analysis” version 3.0. For data acquisition and subsequent analysis of ESI-IT MS data, the software “Trap Control” and “Data Analysis” version 4.1 (all Bruker Daltonics GmbH) were used, respectively.

Statistical analyses were performed using GraphPad Prism 6 (GraphPad Software, San Diego, CA, USA). Mean values and standard deviations were calculated. For the verification of significances nonparametric and two-tailed *t*-tests were performed, and significance was indicated by *p* < 0.05.

## 3. Results and Discussion

Sperm cells are the most diverse cell type on earth, not only regarding their cell morphology [26], but also taxa-specific physiological properties associated with their lipid composition [27]. Reasons for a diverse lipid composition could be the habitat specificity, the diet, the mating frequency, the capability of sperm storage and the mode of fertilization [28]. 

This study characterized sperm lipid compositions of the two particular freshwater fish species *P. motoro* and *A. ruthenus*. These species were selected for the following reasons: (I) while in most cartilaginous fishes the osmolality of the internal body fluids is close to the high osmolality of the marine environment (marine osmoconformers), potamotrygonids as obligate freshwater species [29] are characterized by an osmolality of body fluids typical for bony fishes known to be osmoregulators, regardless of the environmental osmolality [9]. (II) Stingrays possess an ancient mode of internal fertilization, while sturgeons developed an external fertilization mode, but preserved some features in the spermatozoon structure (acrosome) characteristic for internal fertilization in non-fish animals. Among others, the selection of these taxonomically distant species eliminated the effect of different osmotic conditions at which specific lipid patterns of sperm membranes can be formed during spermatogenesis. 

Parts of the present study’s results for stingray sperm are in accordance with another study that investigated the lipid composition of stingray sperm by TLC and GC [14]. However, for GC only those FAs can be detected that are present in the standard mixture. As the standard mixture compositions were different, also the results of the two GC approaches differed but were similar in the amounts of the most abundant FAs in stingray sperm. Furthermore, the relative quantitative analysis of the phospholipids was performed by NMR in the present approach. The results from the NMR approach are not in accordance with the already published TLC data [14]. The reason for this is probably the detection of the TLC spots by charring. This procedure is dependent on the amount of double bonds in the fatty acyl residues of lipids and therefore discriminates between lipids with a lower and a higher amount of double bonds [30]. In contrast to that, NMR as a label-free technique allows for full or relative quantification without any modification of the sample and is more suitable for the quantification of phospholipids [31]. Only the contents of SM (13%) and PI (2.8%) in stingray sperm are comparable between these two approaches. 

### 3.1. Relative Quantitative Differences in Lipid Classes by NMR Measurements

**Figure 1:**
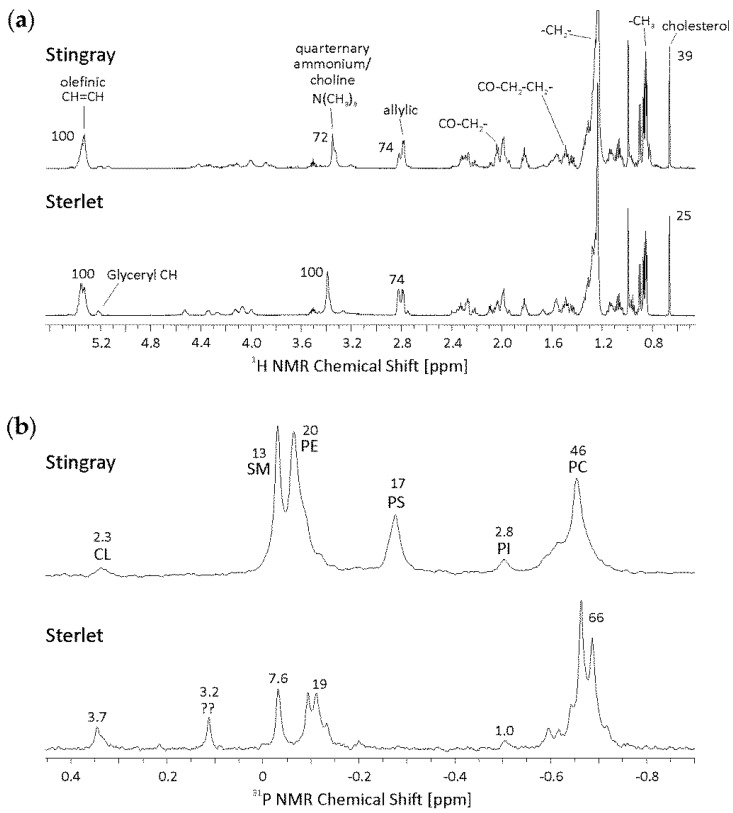
Nuclear magnetic resonance (NMR) spectra of the lipid phase of stingray and sterlet sperm. (**a**) For ^1^H NMR dried lipid extracts were dissolved in 0.5 mL CDCl_3_. Spectra were recorded at 300 K and calibrated by setting the resonance of non-deuterated, residual CHCl_3_ to 7.24 ppm. (**b**) For ^31^P NMR spectra dried lipid extracts were solubilized in sodium cholate buffer (50 mM Tris, 200 mM sodium cholate, 5 mM EDTA, pH 7.65). Spectra were recorded in 5 mm NMR at 310 K. The numbers given represent the relative integral intensities of the different peaks. Only relative data can be obtained because no internal standard was added. Abbreviations: CL—cardiolipin, PC—phosphatidylcholine, PE—phosphatidylethanolamine, PG—phosphatidylglycerol, PI—phosphatidylinositol, PS—phosphatidylserine, SM—sphingomyelin.

Although ^1^H is the most sensitive NMR nucleus, its application to complex mixtures is limited due to the small chemical shift range (about 10 ppm). Thus, the ^1^H NMR spectra of stingray and sterlet look very similar (Figure 1a). However, ^1^H NMR spectra give information about the cholesterol content. Stingray sperm show a higher cholesterol content compared to sterlet sperm (39 to 25 relative integral intensities, respectively) and the relative amount of lipids with a quaternary ammonium group, namely phosphatidylcholine (PC) and sphingomyelin (SM), is higher in sterlet sperm (100 to 72 relative integral intensities). Internal standards were not used in order to avoid potential overlap between the standard and the resonances of interest. The higher amount of cholesterol in stingray sperm could be in accordance with the higher level of SM in the stingray cells. The cholesterol/SM ratio is around 3 in both fish species. This ratio could be crucial for the rigidity of the cell membrane [32] and, thus, for cell function.

Looking at the ^31^P NMR spectra (Figure 1b), differences in the PL compositions of stingray and sterlet sperm are obvious. Sterlet sperm possess a relatively larger moiety of PC than stingray sperm, which is in accordance with the results from the ^1^H NMR experiments. In contrast to stingray sperm, where the phosphatidylserine (PS) resonance is quite prominent, PS is not detectable in sterlet sperm. Furthermore, phosphatidylethanolamine (PE) and PC result in broad, non-resolved peaks in stingray and show more than one resonance in sterlet sperm, although these resonances are not baseline-separated. This is a hint on saturated and unsaturated FAs bound to the glycerol backbone as well as on ether lipids (alkyl-acyl compounds) which are all characterized by slight chemical shift differences. Spectra of sterlet sperm show another, unknown ^31^P resonance at 0.12 ppm which could not be assigned. 

### 3.2. A First Overview about the Lipid Composition by MALDI-TOF MS

**Figure 2:**
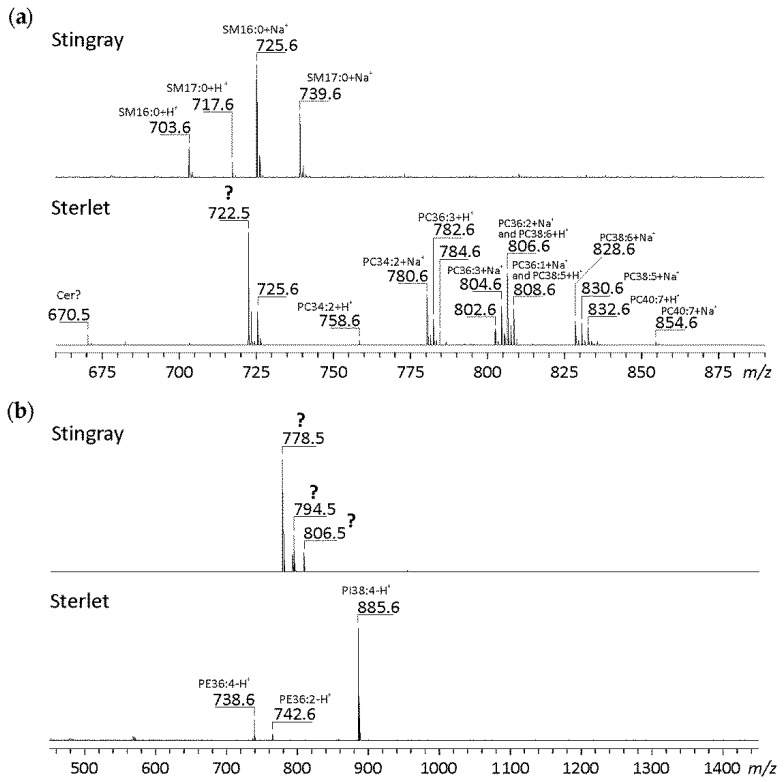
Matrix-assisted laser desorption/ionization time-of-flight (MALDI-TOF) mass spectra of the lipid phase of stingray and sterlet sperm (without previous separation). Spectra were recorded in the positive (**a**) and in the negative ion mode (**b**). In the positive ion mode (phospho)lipids are usually detected as proton (+H^+^) and sodium (+Na^+^) adducts. The structural elucidation of the lipids marked by “?” was performed in this paper (see below).

MALDI-TOF MS is a very sensitive method to detect even traces of lipids. Spectra of crude lipid extracts from stingray and sterlet sperm are shown in Figure 2 and were recorded in the positive (Figure 2a) and in the negative ion mode (Figure 2b). In the positive ion mode, MALDI spectra of PLs usually yield proton (+H^+^, from the acidic matrix) and sodium (+Na^+^) adducts, since Na^+^ is present in all biological samples and cannot be completely removed by the extraction with organic solvents.

Whereas the positive ion mode of the lipid fraction from stingray sperm is dominated by two different SMs, namely SM 16:0 and SM 17:0, many different PC species are detected in sterlet sperm and SM 16:0 is present only in a small amount. This basically agrees with the NMR spectra. Nevertheless, it is remarkable that the presence of SM makes the detection of PC impossible in stingray sperm: since the head groups of both SM and PC are identical, one would expect comparable detection limits for both lipid classes. PC species in sterlet sperm range from those with short-chain FA residues (e.g., PC 34:2) to those with medium-chain (e.g., PC 36:3 and PC 38:6) and long-chain FA residues (e.g., PC 40:7). These differences in chain length and the different degrees of unsaturation are in accordance with the different resonances for PC in the ^31^P-NMR spectrum: the PC fraction of the sterlet is obviously more inhomogeneous compared to the stingray.

Surprisingly, one huge signal is detected in the positive ion spectrum of sterlet sperm at *m/z* 722.5, which cannot be assigned to any common PL. Thus, the structural elucidation of this compound will be performed by other methods (see below). The same is true for the negative ion mode spectrum of stingray sperm. Here, three signals are detected at *m/z* 778.5, 794.5, and 806.5 which do not match any common PL usually detected in the negative ion mode, such as PE, PS, or PI. Since the presence of PS, however, is obvious from the NMR spectra, missing PS peaks in the negative ion mass spectrum indicate the presence of strong electrolytes (such as sulfates) which can suppress less acidic compounds (such as PS or PI). In contrast to this, the negative ion spectrum of sterlet sperm is quite simple and signals can be clearly assigned to two PE and one PI species.

Even though MALDI-TOF MS is a very fast and convenient method for lipid analyses, it needs to be stated that it can only give a crude survey on the lipids to be expected in crude lipid mixtures. Due to ion suppression effects [33], further analyses with more sophisticated methods are necessary. This particularly concerns the chromatographic separation of the lipid mixtures into the individual lipid classes prior to MS analysis.

### 3.3. Lipidomics Studies by Coupling HPTLC to ESI-IT MS

**Figure 3:**
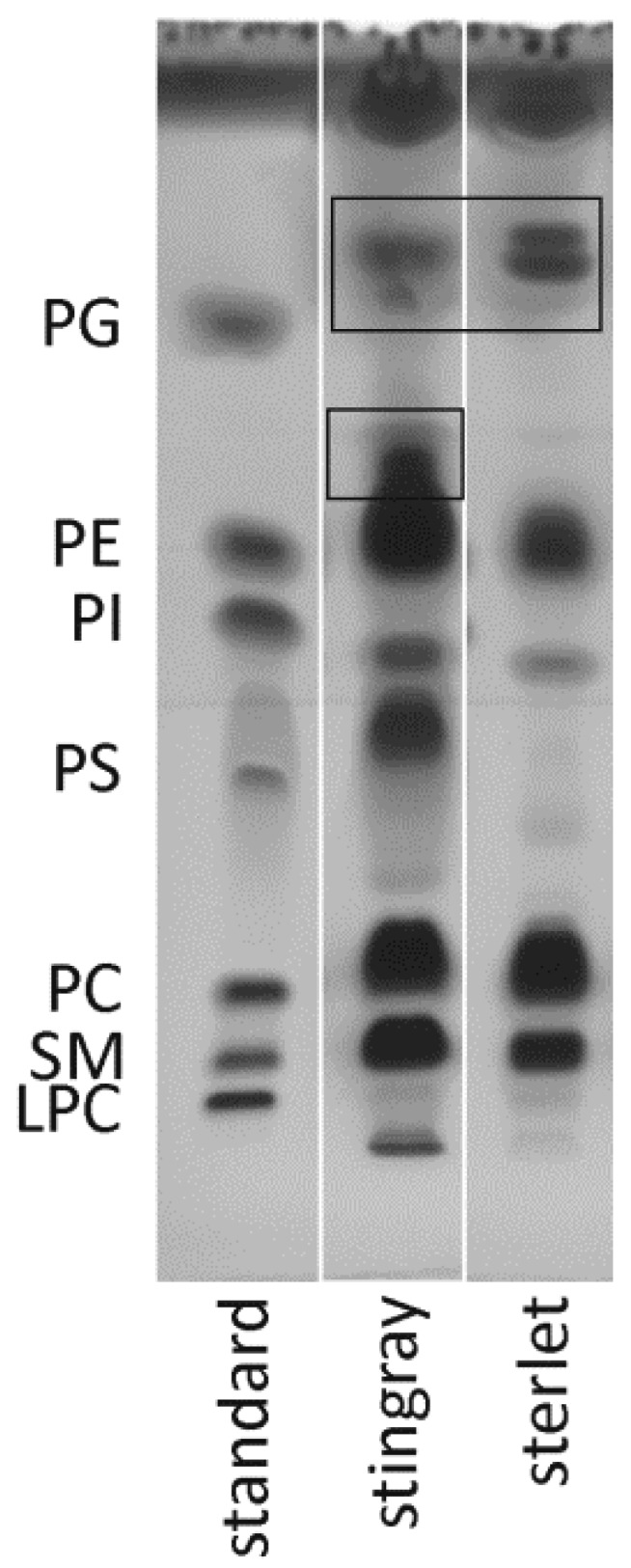
High-performance thin-layer chromatogram of crude lipid extracts of stingray and sterlet sperm. A lipid standard mixture of known composition was applied to the first lane of the glass plate. The chromatograms show clear differences between stingray and sterlet sperm regarding their lipid compositions. Note for instance, the presence of PS in the stingray. Spots of unknown lipid fractions are highlighted by black frames. Abbreviations: LPC—lysophosphatidylcholine, SM—sphingomyelin, PC—phosphatidylcholine, PS—phosphatidylserine, PI—phosphatidylinositol, PE—phosphatidylethanolamine.

HPTLC is a very convenient method for the separation of crude lipid extracts into the different lipid classes. Although HPLC is nowadays more popular, HPTLC is still always widely used and HPTLC plates with “MS grade” purity have been introduced recently [34]. Results of the HPTLC separation show clear differences between stingray and sterlet sperm regarding their lipid compositions (Figure 3). The high abundance of PS in stingray sperm as well as the broad spots of PC and PE is in agreement with the previously discussed ^31^P NMR data. The same applies for the enhanced SM content in the stingray. In contrast to this and to sperm of other externally fertilizing fishes, such as carp, pike, or burbot [16], sterlet sperm do not contain PS, which is very astonishing for several reasons. The PS usually is the most abundant negatively charged phospholipid in membranes of eukaryotic cells, where it has important physiological functions [35]. Furthermore, in mammalian spermatozoa, which possess an acrosome just as sterlet spermatozoa, PS in the plasma membrane and the outer acrosome membrane is necessary for sperm capacitation and acrosome reaction [36]. For avian sperm, a positive correlation between the amount of PS and sperm fertility has been shown [37]. Since there are no data on the sperm phospholipid composition for any other sturgeon species available, it would be premature to suggest the possible reasons for the lack of PS in sterlet sperm established in the present work. Unfortunately, PS analysis and extraction is more difficult compared to other lipids due to its permanent negative charge and the potential loss of PS upon the extraction process [22].

However, there are spots that cannot be assigned to any component of the used PL standard. In stingray sperm, there is a spot above the PE moiety which is absent in the sterlet sample. Both species contain lipids which elute above phosphatidylglycerol (PG, present in the standard), i.e., are characterized by an elevated R_f_ value. The assignments of these spots will be discussed in detail in Section 3.4. It is also important to note that primuline staining provides only relative data because the observed spot intensities depend on (a) the lipid headgroups and (b) the lengths of the fatty acyl residues [38]. 

**Figure A1:**
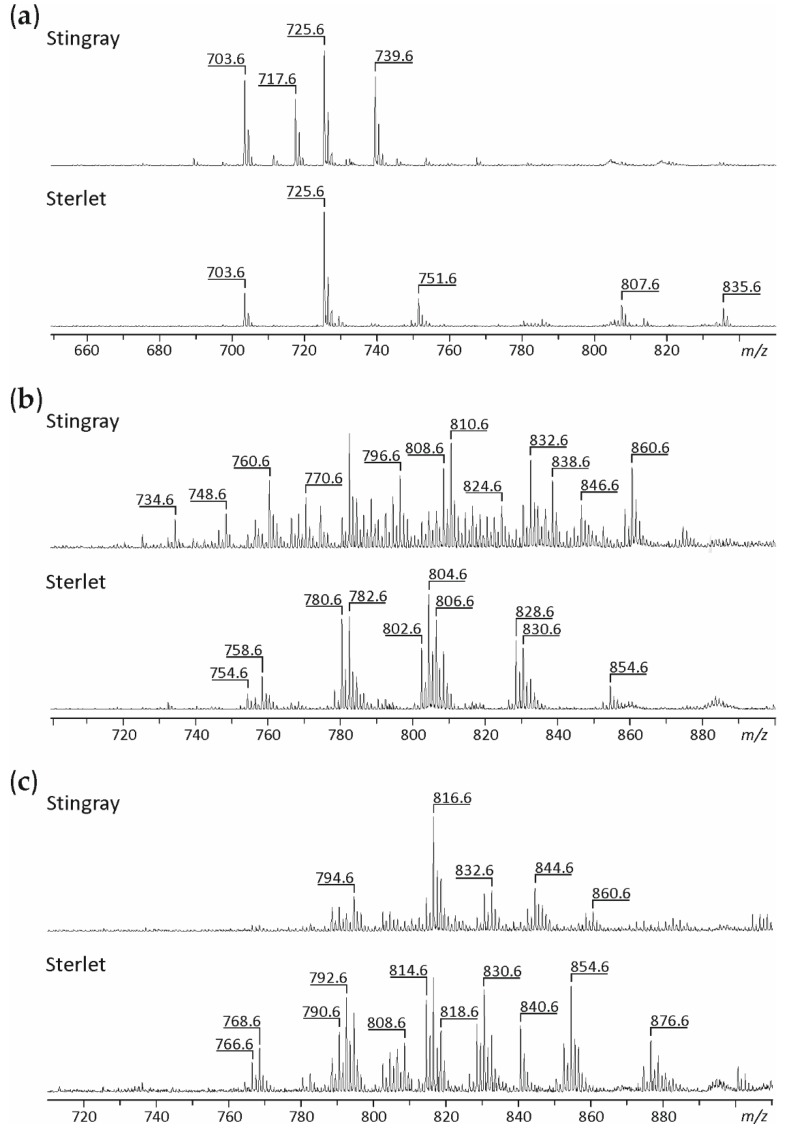
ESI-IT mass spectra of sphingomyelin (SM) and phosphatidylcholine (PC) spots obtained by HPTLC separation. Both fractions were analyzed in the positive ion mode. SM spectra are shown in (**a**). Due to the broad spot of the PC moiety the lower (**b**) and the upper part of the spot (**c**) were analyzed separately. Please see Table A1 for the peak assignment.

**Figure A2:**
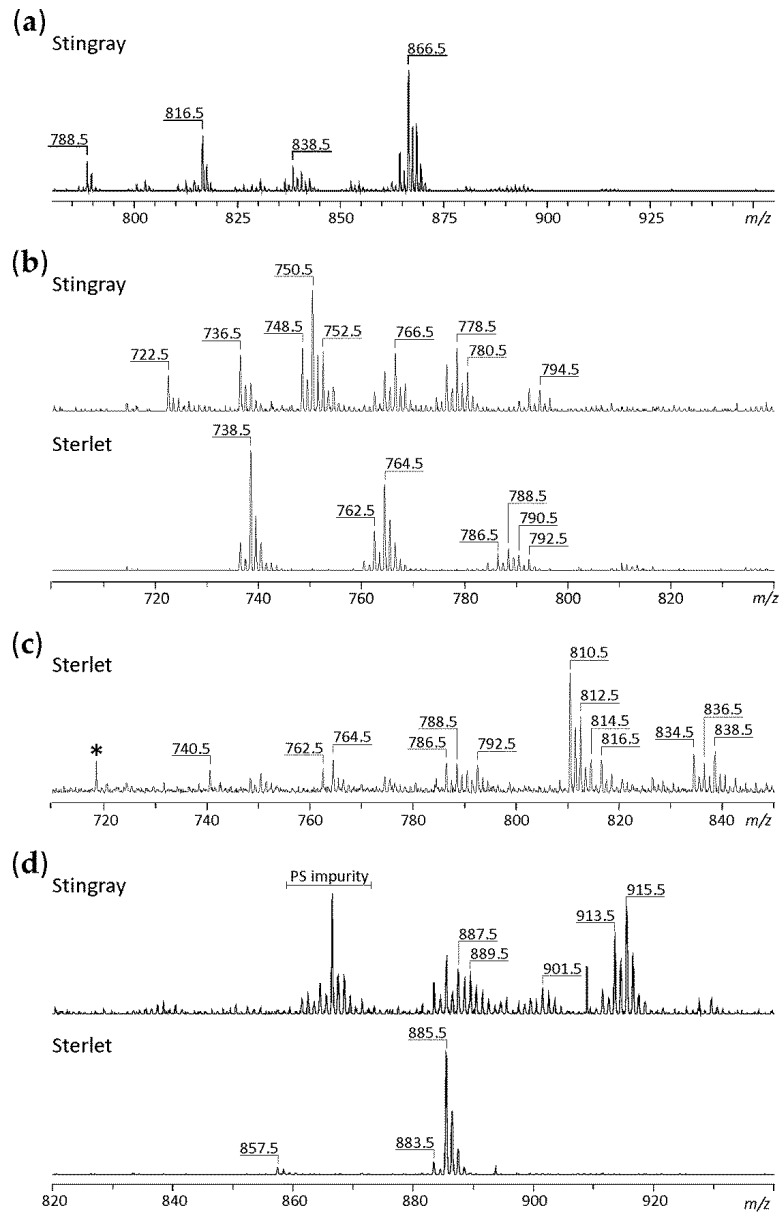
ESI-IT mass spectra of phosphatidylserine (PS), phosphatidylinositol (PI) and phosphatidylethanolamine (PE) spots obtained by HPTLC separation. All fractions were analyzed in the negative ion mode. The spectrum of PS, which was only present in stingray, is shown in (**a**). Due to the broad spot of the PE moiety the lower (**b**) and the upper part of the spot (**c**) were analyzed separately for the sterlet sample. The close proximity of the next spot made a proper analysis of the upper fraction of the stingray PE spot impossible. The spectrum of PI is shown in (**d**). The stingray PI spectrum shows impurities with PS due to the close proximity of both lipid fractions. Please see Table A2 for the peak assignment. * not assigned.

The different TLC spots can be quite easily assigned to the corresponding substances by direct elution of the spots into the ESI mass spectrometer and subsequent ESI MS. The results of the analyses of the SM, PC, PS, PI, and PE spots are depicted in the Appendix A (Figure A1 and Figure A2, Table A1 and Table A2). Even though a resonance in the ^31^P NMR spectra was assigned to cardiolipins (CL), this lipid class has not been investigated in more detail due to its low abundance in the TLC.

In comparison to stingray sperm, sterlet sperm contain SMs with longer chain lengths, such as SM 22:1 and SM 24:1 (Figure A1). 

The PC fractions were divided into a “lower” and an “upper” part (Figure A1) due to the considerable width of the spot on the TLC plate and the knowledge that lipid molecules with shorter chain lengths and acyl-acyl bonds have lower retention factors (R_f_ values) than those with longer chain lengths and alkyl-acyl lipids (ether lipids) [39]. The latter ones are usually characterized by a high extent of unsaturation. Overall, the PC fractions of stingray sperm are comprised of short-chained saturated acyl-acyl lipids (PC 32:0, PC 33:0), of long-chained unsaturated lipids (PC 40:4), and of alkyl-acyl lipids (GPCho o-38:5, GPCho o-40:4). In sterlet sperm PC molecules with even longer acyl chains and even higher degrees of unsaturation could be detected (PC 42:10), but also a huge amount of ether lipids (GPCho o-36:4, GPCho o-38:6). The composition of the unknown lipid spots was also elucidated by TLC-ESI coupling and subsequent MS/MS experiments, which allows the confirmation of putative assignments due to characteristic fragmentation patterns. 

The PS fraction of stingray sperm has a quite simple composition and mainly consists of acyl-acyl species (PS 36:1, PS 38:1, PS 40:4, and PS 42:4, Figure A2). Analysis of the PI spot of stingray resulted in more different PI species and in PI with longer acyl chains (PI 40:3) compared to sterlet sperm which contains nearly exclusively PI 38:4 (Figure A2).

Just as the PC fraction, the PE fraction was also divided into two parts. These analyses showed that the PE fraction of stingray sperm mainly consists of highly unsaturated alkyl-acyl species (GPEtn o-36:5, GPEtn o-38:5, Figure A2). These were not detected in sterlet sperm, which in contrast contains highly unsaturated PE species with medium and long acyl chains (PE 36:4, PE 38:5, PE 42:10, PE 44:12). 

### 3.4. Tandem MS Experiments to Elucidate the Assignments of Unknown Lipid Fractions

**Figure 4:**
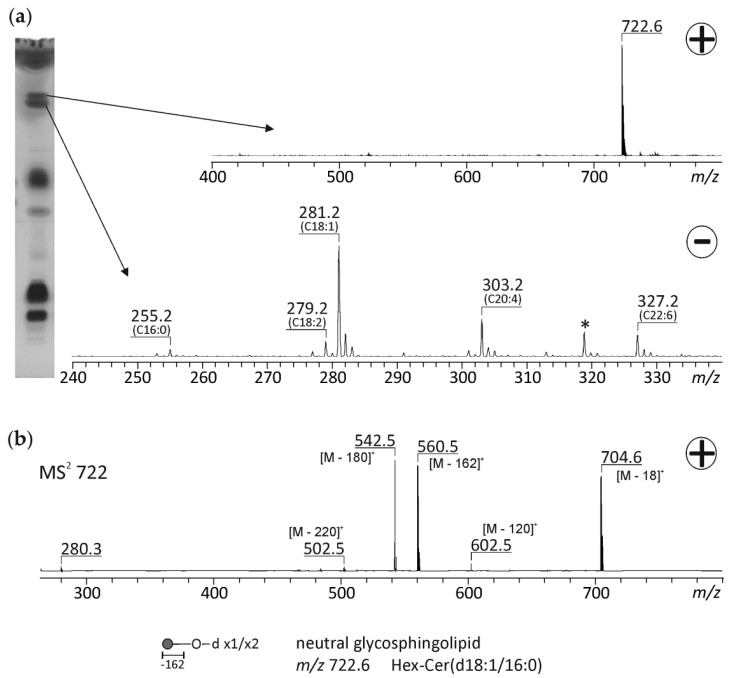
High performance thin-layer chromatography (HPTLC)-electrospray ionization ion trap (ESI-IT) mass spectrometry of the unknown spots of the lipid fraction of sterlet sperm. The chromatographic separation of the lipid extract and the corresponding mass spectra of two unknown spots in the positive and in the negative ion mode, respectively, are shown in (**a**). To further elucidate the structure of *m/z* 722, this ion was subsequently fragmented by collision-induced decay (**b**). Due to characteristic fragments with the loss of a hexose moiety (∆ = 162 and 180) *m/z* 722 was assigned to a neutral glycosphingolipid Hex-Cer(d18:1/16:0). Hex: hexose, Cer: ceramide. * not assigned.

As already mentioned above, there were spots on the HPTLC plate that could not be assigned to any dedicated component of the PL standard, i.e., these compounds represent less common lipids. The results for sterlet are depicted in Figure 4. In sterlet sperm, there are two spots with very similar R_f_ values. The lower of these spots results in peaks in the negative ion mode whose *m/z* values can be assigned to free FAs. The most abundant free FA is oleic acid (C18:1), but also significant amounts of palmitic (C16:0), linoleic (C18:2), arachidonic (C20:4), and docosahexaenoic acid (C22:6) are detected. The high amounts of free FAs detected in sterlet sperm by TLC could account for FAs as substrates for mitochondrial respiration, which are quite diverse in the different externally fertilizing species and have been reviewed recently [40]. The only compound in the upper spot could not be assigned according to its *m/z* value (722.6) and was therefore subjected to MS/MS. The collision-induced decay (CID) spectrum results in distinct cleavage products that can be assigned to characteristic compounds: *m/z* 704 corresponds to water loss from the parent ion. The *m/z* values at 560 and 542 arise from the cleavage of a hexose moiety (Δ = 162) followed by an additional water loss. These characteristic cleavage products allow the assignment of *m/z* 722.6 to a sodiated neutral glycosphingolipid from the simple hexose series, namely Hex-Cer(d18:1/16:0) with the hexose (Hex) being either glucose (Glc) or galactose (Gal). 

**Figure A3:**
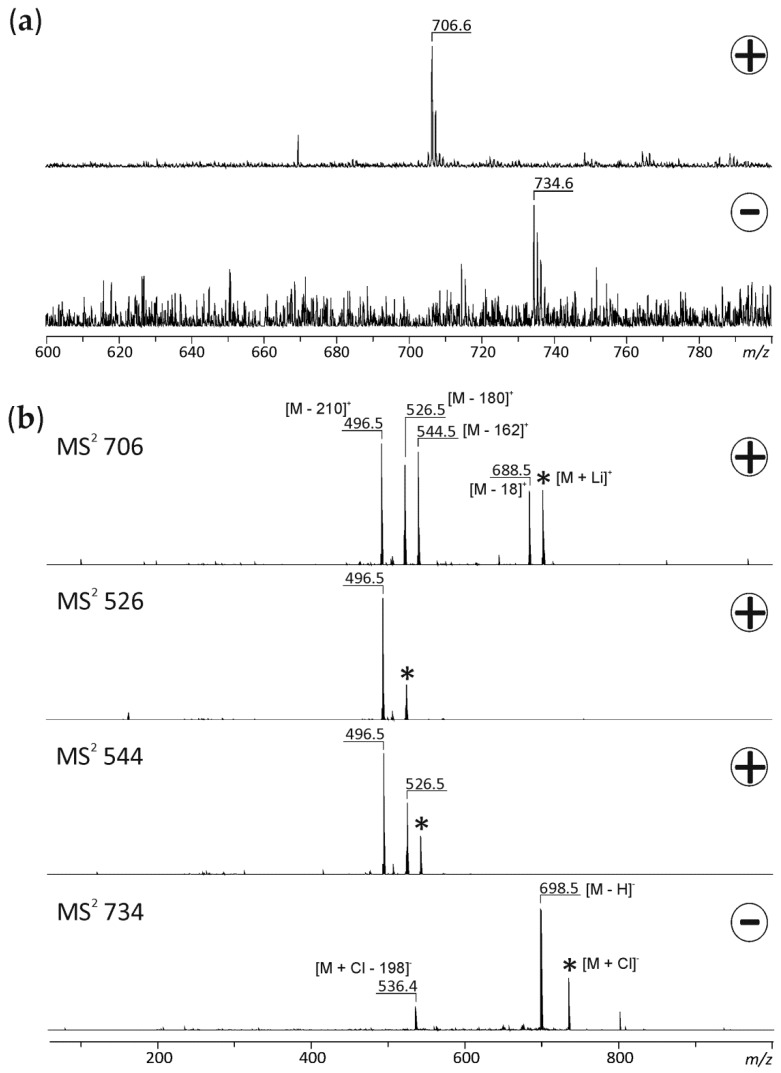
Spectra of ESI-IT MS measurements of sterlet glycosphingolipid back extracted with LiCl. (**a**) The positive ion spectrum shows [M + Li]^+^ at *m/z* 706.6. In the negative ion spectrum *m/z* 734.6 represents [M + Cl]^−^. (**b**) The collision induced dissociation of *m/z* 706 (MS^2^) results in three fragment ions at *m/z* 496, 526, and 544, representing the neutral loss of the sugar moiety ([M + Li – 210]^+^, [M + Li – 180]^+^ and [M + Li – 162]^+^, respectively) and *m/z* 688, representing a water loss ([M + Li – 18]^+^). MS^3^ of *m/z* 526 and 544 also result in *m/z* 496 as the daughter ion. (**c**) The collision-induced dissociation of *m/z* 734 results in *m/z* 698 representing [M – H]^−^ and *m/z* 536 (Δ = 198). * not assigned.

**Figure A4:**
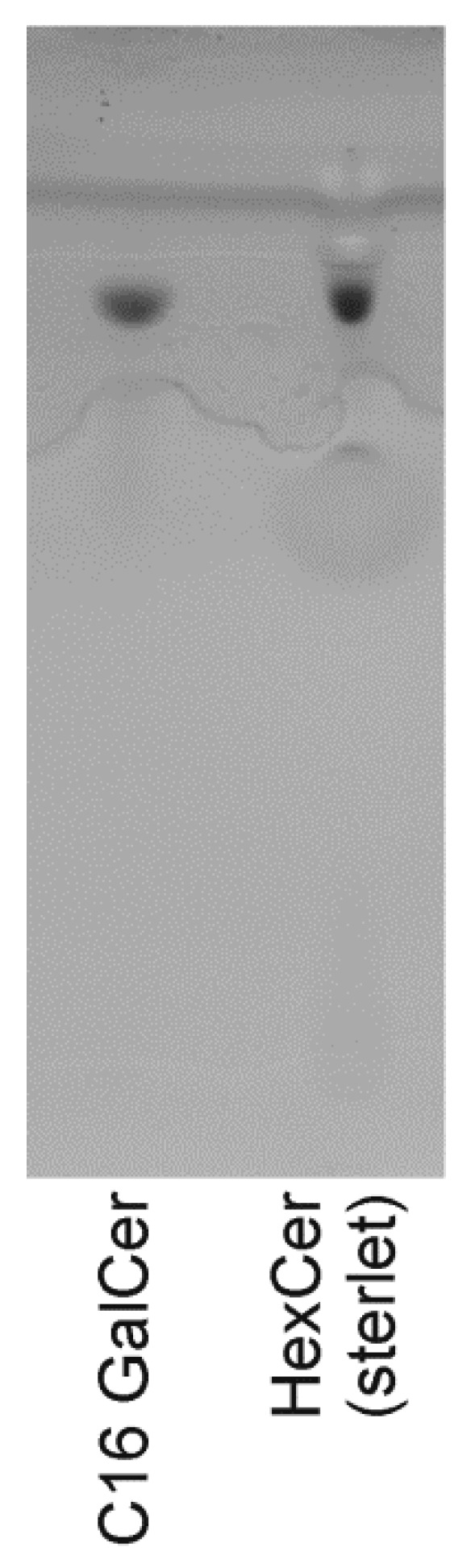
High-performance thin-layer chromatography of glycosphingolipids identified in sterlet sperm extracts. C16 GalCer was applied as standard substance. For methodological details see the main text.

The structure of this molecular species could also be verified by the formation of a lithium adduct (*m/z* 706) according to Han and Cheng [41]. The collisional decay of *m/z* 706 gave rise to positive ions at *m/z* 496.5, 526.5, and 544.5, representing [M + Li – 210]^+^, [M + Li – 180]^+^, and [M + Li – 162]^+^, respectively, due to the neutral loss of the hexose head group (Figure A3). In the negative ion mode the addition of LiCl results in the Cl^−^ adduct, i.e., *m/z* 734.6. The collisional dissociation of this ion resulted in *m/z* 698 [M – Li]^−^, but there were no ions generated at *m/z* 89 and *m/z* 179 (the ratio of which would be useful to distinguish between a galactose and a glucose residue). One potential reason is our use of an ion-trap instrument instead of a quadrupole instrument in the original report. Therefore, a TLC approach was used to differentiate between GalCer and GlcCer [23]. As shown in Figure A4, the Hex-Cer(d18:1/16:0) from sterlet has the same retention time as the standard GalCer. Therefore, the hexose moiety in this neutral glycosphingolipid can be assigned to galactose. This GalCer(d18:1/16:0) could be crucial for sperm–egg interaction in sterlet. The glycolipid composition of sperm from externally fertilizing fishes might also be dependent on the spawning environment. 

**Figure 5:**
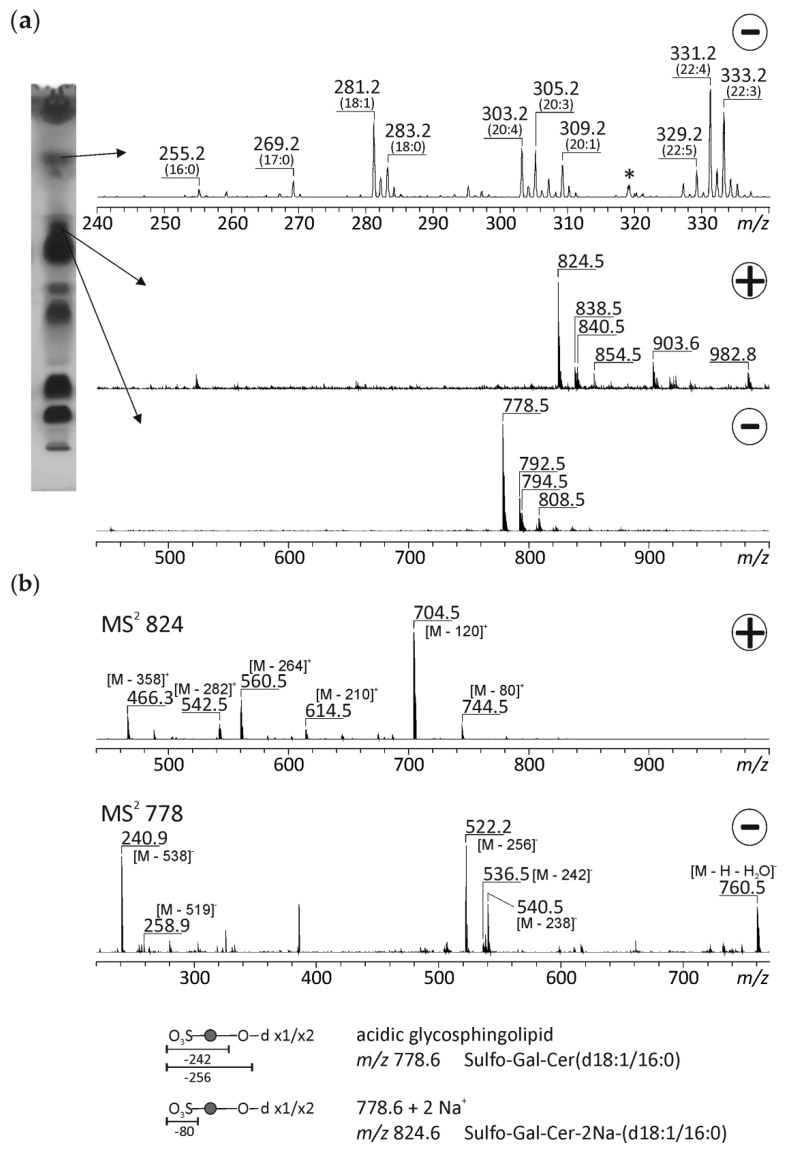
HPTLC-ESI-IT MS of unknown spots of the lipid fraction of stingray sperm. The chromatographic separation of the lipid extract and the corresponding positive and negative ion mass spectra of two unidentified spots are shown in (**a**). To further elucidate the structure of *m/z* 824 in the positive and *m/z* 778 in the negative ion mode, these ions were fragmented by collision-induced decay (**b**). Due to the detectability in the positive and the negative ion mode and the characteristic fragments with the loss of a sulfate group (∆ = 80) and the detection of a sulfated sugar moiety in the negative ion mode (*m/z* 259 and 241), both signals were assigned to an acidic glycosphingolipid sulpho-Gal-Cer(d18:1/16:0). The signals at *m/z* 838/792, 840/794, and 854/808 could be putatively assigned to sulpho-Gal-Cer(d18:1/17:0), sulpho-Gal-Cer(d18:0/17:0), and sulpho-Gal-Cer(d18:0/18:0), respectively. Gal—galactose, Cer—ceramide. The assignment of the hexose unit to galactose was made due to the characteristic biosynthesis pathway of sulfo glycolipids [51]. * not assigned.

Stingray sperm also contain free FAs (Figure 5). However, the composition is significantly different from sterlet sperm. In stingray, the most abundant free FA is docosatetraenoic acid (C22:4), but also huge amounts of docosatrienoic acid (C22:3), arachidonic acid, eicosatrienoic acid (C20:3), and oleic acid are present. In particular C22:4 and C22:3 are not detectable at all in sterlet sperm. Analyses of the spot with a slightly higher R_f_ value than the PE in stingray sperm gave rise to *m/z* 824.5 as the most abundant ion in the positive and *m/z* 778.5 in the negative ion mode. The CID spectrum of *m/z* 824.5 reveals a mass loss of 80, which is characteristic for a sulfate group, plus the same compounds that can be detected in the case of sterlet at *m/z* 722.5, i.e., both compounds have similar structures. The fragmentation of *m/z* 778 resulted in losses of *m/z* 242 and 256, which are characteristic of a sulfated hexose moiety [42,43]. It is known that sulfated glycolipids can be basically detected in the positive and in the negative ion mode [44]. Therefore, both signals (*m/z* 824.5 and 778.5) were assigned to the same molecule, namely the acidic glycosphingolipid sulpho-Gal-Cer(d18:1/16:0). Sulfoglycosphingolipids have already been found in avian sperm [45]. A specific function of the sulfatides in mediating interactions between spermatozoa and components of the female genital duct was assumed for birds, which are known to store male gametes in female reproductive ducts [45]. Female stingrays are also assumed to store sperm for a prolonged period [46], like other cartilaginous fishes which can store sperm for a year and beyond [47,48]. In mammals, the sulfogalactosylglycerolipid, called seminolipid, is indispensable for spermatogenesis, sperm function, and fertilization (reviewed in [49]). Specifically, the involvement of seminolipid in the direct binding to the zona pellucida was suggested [50]. Seminolipid was found neither in stingray nor sterlet sperm. Despite these results, the presence and function of glycolipids in fish sperm is not well understood and needs further investigation.

### 3.5. Gas Chromatographic Investigations of the Lipid Fraction

The overall FA composition of the stingray and sterlet sperm lipids was investigated by means of GC. The results are summarized in Table 1. Even though the total amount of saturated FAs (SFAs) is nearly identical in stingray and sterlet sperm, there were some characteristic compositional differences. In stingray sperm, there is significantly more C17:0 and C18:0, but significantly less C16:0. The total amount of monounsaturated FAs (MUFAs) is significantly higher in stingray sperm. This is based particularly on much higher moieties of C17:1cis-9, C18:1cis-11, and C20:1cis-11. The content of C18:1cis-9 (oleic acid), however, is significantly lower in stingray compared to sterlet sperm. There is increasing evidence that *trans* FAs possess significant biological relevance [52]. However, these compounds were not further investigated because of two reasons: (a) only very small amounts of *trans* FAs were observed in the samples and (b) the used GC reference mixture is not the optimum one to detect *trans* FAs. Nevertheless, *trans* FAs will be considered in future experiments because they may play a major role in sperm physiology and particularly in oxidation processes.

**Figure A5:**
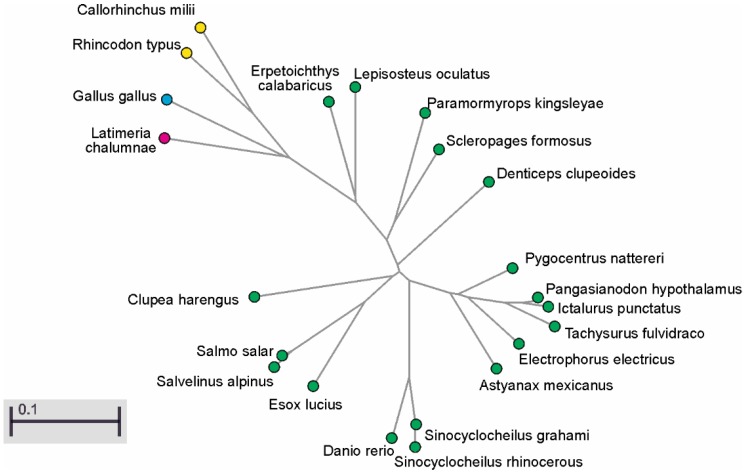
Phylogenetic tree of the elongation of very long chain fatty acids protein (ELOVL) 2 in chicken (*Gallus gallus*, blue spot), cartilaginous fishes (*Callorhinchus millii* and *Rhincodon typus*, yellow spots), the coelacanth *Latimeria calumnae* (pink spot), and bony fishes (green spots). The search for similarities between the protein sequences was performed online by the Constraint-based Multiple Alignment Tool of the U.S. National Centre of Biotechnology Information (https://www.ncbi.nlm.nih.gov/tools/cobalt/). The tree was calculated by the algorithm Fast Minimum Evolution. The evolutionary distance between the sequences was modeled by the Grishin distance model.

**Figure A6:**
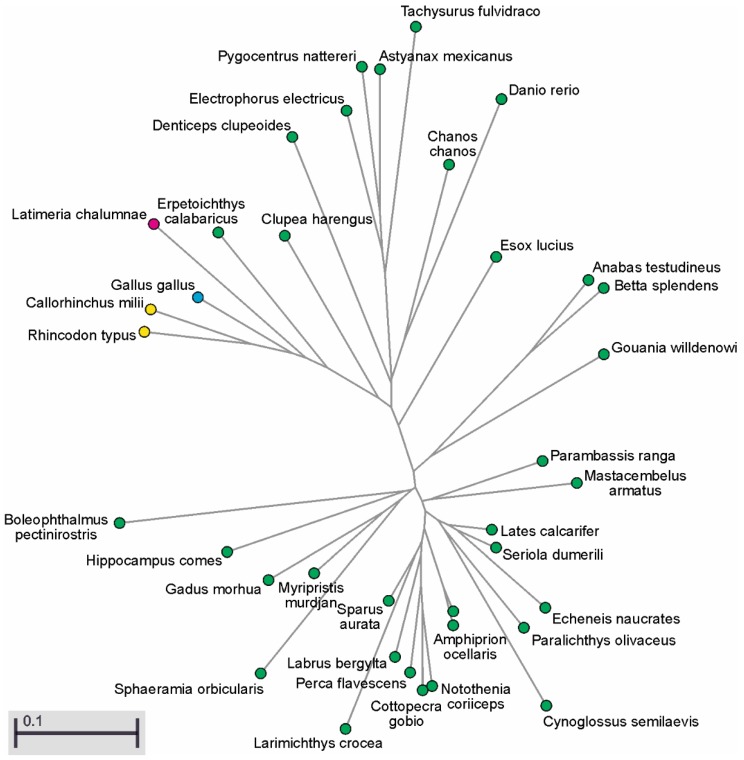
Phylogenetic tree of the elongation of the protein 6-desaturase in chicken (*Gallus gallus*, blue spot), cartilaginous fishes (*Callorhinchus millii* and *Rhinocodon typus*, yellow spot), the coelacanth *Latimeria calumnae* (pink spot) and bony fishes (green spots). The search for similarities between the protein sequences was performed online by the Constraint-based Multiple Alignment Tool of the U.S. National Centre of Biotechnology Information (https://www.ncbi.nlm.nih.gov/tools/cobalt/). The tree was calculated by the algorithm Fast Minimum Evolution. The evolutionary distance between the sequences was modeled by the Grishin distance model.

In contrast to stingray sperm, sterlet sperm contains a significantly higher amount of n-3 polyunsaturated FAs (PUFAs)—particularly C22:6n-3 (docosahexaenoic acid). Whereas C18:2n-6 was more than 10 times higher abundant in sterlet sperm, C22:4n-6 was more than 20 times elevated in stingray sperm. In this case, stingray sperm is similar to avian sperm. High amounts of C20:4 and C22:4 and low amounts of C22:6 have also been described for pheasant and chicken sperm [37,45,53]. A positive correlation between fertility and the levels of C20:4n-6 and C22:4n-6 in chickens was reported [37]. C22:4n-6 was so far only found in low concentrations in externally fertilizing fishes [16]. Dedicated functions of C22:4n-6 have not been described, so far. It is only known that it is an elongation product of C20:4n-6 and an educt for C24:4n-6, C24:5n-6, and C22:5n-6 [54]. Due to low concentrations of the latter ones, a further conversion of C22:4n-6 might not play a significant role in stingray sperm. One explanation for the different fatty acids might be a low enzymatic activity of the respective fatty acyl elongases and desaturases. The search for similarities between protein sequences of fatty acyl elongases and desaturases by the basic local alignment search tool (BLAST) revealed close proximities of the elongation of very long chain fatty acids protein (ELOVL) 1, 2, 5, and 6 as well as close proximities of the fatty acyl desaturases 4, 5, and 6 between birds and cartilaginous fishes (exemplarily depicted for ELOVL2 and 6-desaturase in Figure A5 and Figure A6). C22:4n-6 may play a role during the fertilization process of the oocyte.

In sterlet sperm the amount of C20:4n-6 was also high, but C18:2n-6 was more than 10 times higher and C22:6n-3 was more than four times higher compared to stingray sperm. This substantiates the results of the HPTLC-ESI-MS analyses (Section 3.3), where high amounts of putative C22:6-containing phospholipids (e.g., PC 42:10, PE 44:12) were detected. The ratio of n-3 and n-6 PUFAs was considerably higher in sterlet sperm (0.71) compared to stingray sperm (0.41). High amounts of n-3 FAs have already been reported for other externally fertilizing fish species [16,55,56,57]. Despite these differences, the ratio SFA/(MUFA+PUFA) was nearly identical in sterlet and stingray sperm (sterlet: 0.24, stingray: 0.25). The PUFA/MUFA ratio of sterlet sperm was comparable to rainbow trout and burbot, but lower compared to pike and carp [15,16] and only marginally higher compared to stingray. 

## 4. Conclusions

This study provides novel data on sperm lipid compositions and lipid contents for freshwater stingray and sterlet characterized by two different modes of fertilization. Generally, marked differences in the composition and the content of lipids known to be involved in membrane properties, signaling, and bioenergetic supply of motility were clearly detected for sperm cells of both fish species. Nonetheless, inasmuch as it is the first comprehensive description of sperm lipid composition for representatives of cartilaginous fishes and acipenserids (possessing internal and external modes of fertilization, respectively), further studies on other representatives of cartilaginous fishes and acipenserids are needed to relate the found differences with taxonomic positions and/or the fertilization mode of these fish groups. 

## Figures and Tables

**Table 1:** Fatty acid composition of stingray and sterlet sperm lipids. Data are given in % of total fatty acids. Abbreviations: LA—linoleic acid, LNA—linolenic acid, CLA—conjugated linoleic acid, AA—arachidonic acid, EPA—eicosapentaenoic acid, ADA—adrenic acid, DPA—docosapentaenoic acid, DHA – docosahexaenoic acid. * *p* < 0.5, ** *p* < 0.01, *** *p* < 0.001.

	Sterlet (*n* = 4)		Stingray (*n* = 5)	
	Mean	SD	Mean	SD
**Saturated Fatty Acids (SFA)**				
C8:0	<0.01	-	0.09	0.16
C10:0	0.17	0.06	0.15	0.05
C12:0	1.37	1.22	0.78	0.17
C13:0	0.02	0.03	<0.01	-
C14:0	0.49	0.36	0.38	0.20
C15:0	0.02	0.01	0.01	0.01
C16:0	13.70	1.80	6.48 *	0.62
C17:0	0.22	0.11	1.32 ***	0.14
C18:0	2.29	0.87	8.99 **	0.27
C20:0	0.05	0.02	0.09	0.02
C21:0	<0.01	-	0.09	0.11
C22:0	0.17	0.21	1.11	0.15
C23:0	0.49	0.63	0.28	0.21
C24:0	0.05	0.04	<0.01	-
**Sum SFA**	**19.05**	**5.37**	**19.76**	**2.12**
**Monounsaturated Fatty Acids (MUFA)**				
C16:1cis-9	1.36	0.08	1.93 **	0.16
C17:1cis-9	0.05	0.05	2.85 ***	0.26
C18:1trans-9	0.03	0.00	0.17	0.16
C18:1trans-11	<0.01	-	0.06	0.03
C18:1cis-9	15.94	2.07	5.61 *	0.50
C18:1cis-11	1.55	0.07	6.24 ***	0.67
C20:1cis-11	0.84	0.04	7.34 ***	0.43
C22:1cis-13	0.09	0.01	0.14 **	0.01
C24:1cis-15	0.08	0.01	<0.01	-
**Sum MUFA**	**19.94**	**2.33**	24.34 *	**2.21**
**Polyunsaturated Fatty Acids (PUFA)**				
C18:2 trans-9,trans-12	0.06	0.06	0.09	0.06
C18:2n-6 (LA)	6.95	1.55	0.63 *	0.09
C18:3n-6	1.10	0.09	0.09 ***	0.05
C18:3n-3 (LNA)	0.41	0.07	0.26 *	0.04
C18:2cis-9,trans-11 (CLA)	<0.01	-	0.02	0.01
C18:4n-3	0.14	0.03	0.44	0.52
C20:2n-6	0.65	0.02	0.21 ***	0.03
C20:3n-6	2.38	0.27	1.44 **	0.31
C20:3n-3	0.08	0.03	<0.01	-
C20:4n-6 (AA)	22.42	1.76	18.49 *	1.86
C22:2n-6	0.08	0.01	0.05 *	0.01
C20:5n-3 (EPA)	8.75	1.76	9.61	0.89
C22:4n-6 (ADA)	0.63	0.09	16.84 **	3.56
C22:5n-6	2.03	0.62	2.18	0.34
C22:5n-3 (DPA)	1.39	0.20	2.68 **	0.38
C22:6n-3 (DHA)	14.01	1.12	3.20 ***	1.19
**Sum PUFA**	**61.09**	**7.69**	**56.23**	**9.33**
**Sum n-3 PUFA**	**24.80**	**3.21**	16.18 **	**3.01**
**Sum n-6 PUFA**	**35.13**	**4.32**	**39.85**	**6.19**

## Appendix A

**Table A1:** Assignment of peaks detected in the sphingomyelin (SM) and the phosphatidylcholine (PC) spots by ESI-IT MS after HPTLC separation of crude lipid extracts from stingray and sterlet sperm. Spectra were recorded in the positive ion mode and both lipid classes were detected as proton (+ H^+^) and sodium (+ Na^+^) adducts.

Sphingomyelins	
*m*/*z*	Assignment
703.6	SM 16:0 + H^+^
717.6	SM 17:0 + H^+^
725.6	SM 16:0 + Na^+^
739.6	SM 17:0 + Na^+^
751.6	SM 18:1 + Na^+^
807.6	SM 22:1 + Na^+^
835.6	SM 24:1 + Na^+^
**Phosphatidylcholines (Lower Fraction)**	
734.6	PC 32:0 + H^+^
748.6	PC 33:0 + H^+^
754.6	PC 32:1 + Na^+^
756.6	PC 32:0 + Na^+^
758.6	PC 34:2 + H^+^
760.6	PC 34:1 + H^+^
770.6	PC 33:0 + Na^+^
774.6	PC 35:1 + H^+^
780.6	PC 34:2 + Na^+^
782.6	PC 34:1 + Na^+^
784.6	PC 34:0 + Na^+^/PC 36:3 + H^+^
786.6	PC 36:2 + H^+^
788.6	PC o-36:5 + Na^+^/PC 36:1 + H^+^
792.6	PC o-36:3 + Na^+^
794.6	PC o-38:5 + H^+^
796.6	PC 35:1 + Na^+^
802.6	PC 36:5 + Na^+^
804.6	PC 36:4 + Na^+^
806.6	PC 36:3 + Na^+^
808.6	PC 36:2 + Na^+^/PC 38:5 + H^+^
810.6	PC 38:4 + H^+^
816.6	PC o-38:5 + Na^+^/PC 38:1 + H^+^
824.6	PC o-38:1 + Na+/PC o-40:4 + H^+^
828.6	PC 38:6 + Na^+^
830.6	PC 38:5 + Na^+^
832.6	PC 38:4 + Na^+^
836.6	PC 40:5 + H^+^
838.6	PC 38:1 + Na^+^/PC 40:4 + H^+^
846.6	PC o-40:4 + Na^+^
854.6	PC 40:7 + Na^+^
858.6	PC 40:5 + Na^+^
860.6	PC 40:4 + Na^+^
**Phosphatidylcholines (Upper Fraction)**	
766.6	PC o-36:5 + H^+^
768.6	PC o-36:4 + H^+^
788.6	PC o-36:5 + Na^+^
790.6	PC o-36:4 + Na^+^
792.6	PC o-38:6 + H^+^
794.6	PC o-38:5 + H^+^
802.6	PC 36:5 + Na^+^
804.6	PC 36:4 + Na^+^
806.8	PC 38:6 + H^+^
808.6	PC 38:5 + H^+^
814.6	PC o-38:6 + Na^+^
816.6	PC o-38:5 + Na^+^
818.6	PC o-38:4 + Na^+^/PC p-40:6 + H^+^
828.6	PC 38:6 + Na^+^
830.6	PC 38:5 + Na^+^/PC 40:8 + H^+^
832.6	PC 38:4 + Na^+^/PC 40:7 + H^+^
840.6	PC p-40:6 + Na^+^
844.6	PC o-40:5 + Na^+^
852.6	PC 40:8 + Na^+^/PC 42:11 + H^+^
854.6	PC 40:7 + Na^+^/PC 42:10 + H^+^
856.6	PC 40:6 + H^+^/PC 42:9 + H^+^
860.6	PC 40:4 + Na^+^
874.6	PC 42:11 + Na^+^
876.6	PC 42:10 + Na^+^
878.6	PC 42:9 + Na^+^

**Table A2:** Assignment of peaks detected in phosphatidylserine (PS), phosphatidylethanolamine (PE), and phosphatidylinositol (PI) spots by ESI-IT MS after HPTLC separation of crude lipid extracts from stingray and sterlet sperm. Spectra were recorded in the negative ion mode and both lipid classes were detected as deprotonated species (−H^+^).

**Phosphatidylserines**	
788.5	PS 36:1 – H^+^
816.5	PS 38:1 – H^+^
838.5	PS 40:4 – H^+^
840.5	PS 40:3 – H^+^
864.5	PS 42:5 – H^+^
866.5	PS 42:4 – H^+^
868.5	PS 42:3 – H^+^
**Phosphatidylethanolamines (Lower Fraction)**	
722.5	PE o-36:5 – H^+^
736.5	PE 36:5 – H^+^
738.5	PE 36:4 – H^+^
748.5	PE o-38:6 – H^+^
750.5	PE o-38:5 – H^+^
752.5	PE o-38:4 – H^+^
754.5	PE o-38:3 – H^+^
762.5	PE 38:6 – H^+^
764.5	PE 38:5 – H^+^
766.5	PE 38:4 – H^+^
768.5	PE 38:3 – H^+^
776.5	PE o-40:6 – H^+^
778.5	PE o-40:5 – H^+^
780.5	PE o-40:4 – H^+^
786.5	PE 40:8 – H^+^
788.5	PE 40:7 – H^+^
790.5	PE 40:6 – H^+^
792.5	PE 40:5 – H^+^
794.5	PE 40:4 – H^+^
796.5	PE 40:3 – H^+^
**Phosphatidylethanolamines (Upper Fraction)**	
740.5	PE 36:3 – H^+^
762.5	PE 38:6 – H^+^
764.5	PE 38:5 – H^+^
786.5	PE 40:8 – H^+^
788.5	PE 40:7 – H^+^
792.5	PE 40:5 – H^+^
810.5	PE 42:10 – H^+^
812.5	PE 42:9 – H^+^
814.5	PE 42:8 – H^+^
816.5	PE 42:7 – H^+^
834.5	PE 44:12 – H^+^
836.5	PE 44:11 – H^+^
838.5	PE 44:10 – H^+^
**Phosphatidylinositols**	
857.5	PI 36:4 – H^+^
883.5	PI 38:5 – H^+^
885.5	PI 38:4 – H^+^
887.5	PI 38:3 – H^+^
889.5	PI 38:2 – H^+^
901.5	PI 39:3 – H^+^
911.5	PI 40:5 – H^+^
913.5	PI 40:4 – H^+^
915.5	PI 40:3 – H^+^
